# Rapid determination of paeoniflorin from *Paeonia sinjiang K. Y. Pan*. by rapid resolution liquid chromatography

**DOI:** 10.4103/0973-1296.62893

**Published:** 2010-05-05

**Authors:** Xiaoying Zhou, Xiao-Ming Chen, Liang Ge, Hai-Yan Gong, Shuge Tian, Dongqing An

**Affiliations:** 1 *Xinjiang Key Laboratory of Famous Prescription and Science of Formulas, Urumqi - 830 011, China*; 2 *College of Pharmacy, XinJiang Medical University, Urumqi - 830 011, China*; 3 *Strathclyde Institute of Pharmacy and Biomedical Sciences, University of Strathclyde, Scotland-G1 1QE, United Kingdom*

**Keywords:** *Paeonia sinjiang K. Y. Pan*, paeoniflorin, rapid resolution liquid chromatography

## Abstract

A rapid, effective, binary reverse phase rapid resolution liquid chromatographic method has been developed for the determination of Paeoniflorin extracted from *Paeonia sinjiang K. Y. Pan*. with short run time. RRLC separation was achieved by using Agilent (Zorbas XDB-C_18_ 4.6 mm × 50 mm, 1.8 *μ*m) column with mobile phase complosed of methanol and 0.05 mol/l potassium phosphate monobasic. Flow rate was at 1.0 ml/min. Retention time was at about 1.16 min. Recovery rate was 99.8% and relative standard deviation of three replicate samples tested was 1.8%. RRLC method has been applied to both *in vitro* studies of paeoniflorin formulation and drug estimation in biological samples.

## INTRODUCTION

**Figure 1:**
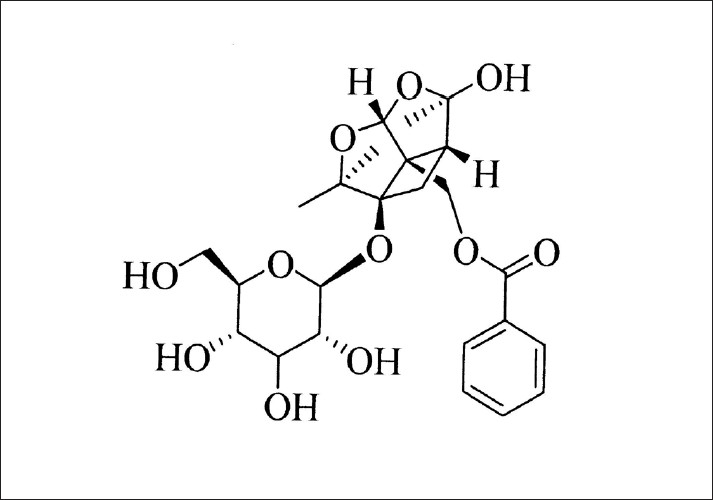
Chemical formula of paeoniflorin

*Paeonia sinjiang K. Y. Pan*. root is a very important drug substance in Chinese herbal medicine for heat-clearing, blood-cooling, activating blood, absorbing clots and eliminating carbuncle.[1] This traditional Chinese herbal medicine has been widely used and studied for its pharmacological and clinical application, e.g. one of its functions of inhibiting aggregation of platelet,[2] and stimulating hepatic cell regeneration,[3,4] stabilizing erythrocyte membrane structure,[5] removing thrombus, preventing coagulation,[6] avoiding hepatic fibrosis,[7] stopping atherosclerosis, protecting heart and liver and anti tumor,[8] etc. *Paeonia sinjiang K. Y. Pan*. contains plentiful of glycoside compounds, one of the dominant component is paeoniflorin which makes up between 3.5% and 7.98% by weight of this herbal medicine[9] [Figure 1].

Paeoniflorin[5beta-[(Benzoyloxy)methyl]tetrahydro-5-hydroxy-2-methyl-2,5methano-1H-3,4-dioxacyclobuta[cd]pentalen-1alpha (2H)-yl-beta-D-glucopyranoside] is the major white substance exacted from *Paeonia sinjiang K. Y. Pan*., its chemical structures presented as in Figure 1. Paeoniflorin is a naturally existing monoterpenes photochemical compound currently being studied in traditional Chinese medicine for its spasmolysis, analgesia, sedation and anticonvulsant efficacy.[10] In previous researches, many HPLC methods have been developed for quantitating paeoniflorin concentration in biological samples.[11–14] Besides, there was another quantitative method developed using high performance liquid chromatography with DAD as detector and coupled with electro spray ionization-mass spectrometry (HPLC-DAD/ESI-MS).[15,16] It is necessary to follow up the study and further develop this traditional Chinese herbal medicine. With the possibility of further discovery and determination of new chemical components in *Paeonia sinjiang K. Y. Pan*.,[17,18] and the research of their pharmacological actions,[18,19] more and more Chinese researchers are using modern technologies to analyze the traditional Chinese medicine, *Paeonia sinjiang K. Y. Pan*, and probably it may have broad application prospects.

## MATERIALS AND METHODS

### Plant materials and reagents

*Paeonia sinjiangensis K.Y.Pan*. was collected from Altai area in Xinjiang Province, China. Plant materials are further identified by Yonghe Li, a pharmacist from Chinese medicine hospital of Xinjiang. These dried specimens were kept in the Traditional Chinese Medicine College demonstration room of Chinese herbal samples at Xinjiang Medical University.

Paeoniflorin (pKa = 4.4) standard reference was obtained from National Institute for The Control of Pharmaceutical and Biological Products, NO: 110736-200423. HPLC grade methanol was supplied by USA, Fisher Scientific, NO: 201-796-7100. Potassium phosphate monobasic of analytical grade was provided by Tianjin TianXin Chemical Reagents Company. All sample solutions and buffers were prepared with water made by Millipore Q3 ultra-pure water system (Millipore, USA) Agilent.

### Instrument and chromatographic conditions

The RRLC system consists of 1200SL RRLC (Agilent, USA, with Zorbas XDB-C_18_ column 4.6 mm×50 mm, 1.8 μm), and photodiode array detector. The system was equipped with Thermos tatted Column Compartment and Auto samplers and Micro Well plate Auto sampler. Operation of the RRLC separation and the data processing of RRLC system were performed by the Cessation for LC 3D Systems software. RRLC separations were achieved using a Zorbas XDB-C_18_ column and a guard column. Mobile phase was made up of methanol and 0.05 mol/l potassium phosphate monobasic buffer at the volume ratio of 38:62, mobile phase was filtered through a 0.45 μm thick membrane and degassed by ultrasonication under vacuum state before use. Flow rate was maintained at 1.0 ml/min and samples were monitored with UV detector at the wave length of 230 nm. Injection volume was set at 5 μl. All RRLC experiments were run at 30°C.

### Standard preparation

A total of 0.5 mg/ml of Paeoniflorin standard solution was prepared by dissolving Paeoniflorin standard reference in HPLC grade methanol.

### Sample solution preparation

Stock solutions were prepared by dissolving 0.5 g of paeoniflorin extracted from *Paeonia sinjiangensis K.Y.Pan*, then dissolved in 25 ml HPLC grade methanol to obtain 20 mg/ml. Paeoniflorin standard reference solutions for linearity quantification were prepared by diluting the stock solution with mobile phase consisted of 38% methanol and 62% 0.05 mol/l potassium phosphate monobasic buffer immediately before use. All the preparations were progressed in borosilicate glass tubes.

## RESULTS

Determination of Paeoniflorin Content in *Paeonia sinjiang K. Y. Pan*.

**Figure 2:**
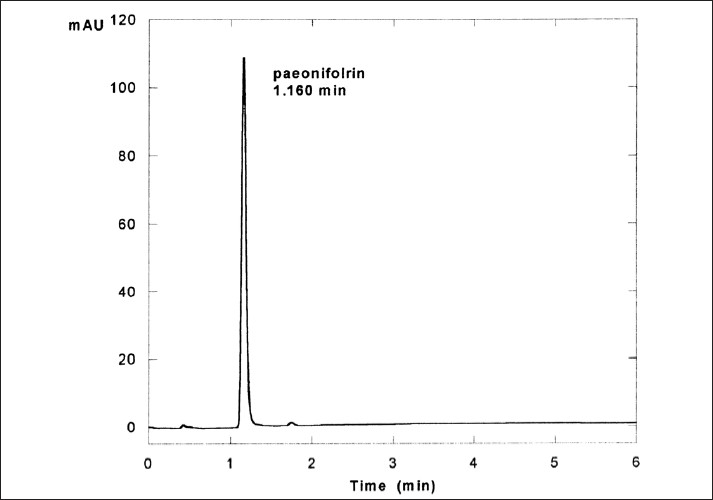
Typical RRLC chromatogram of paeoniflorin

**Figure 3:**
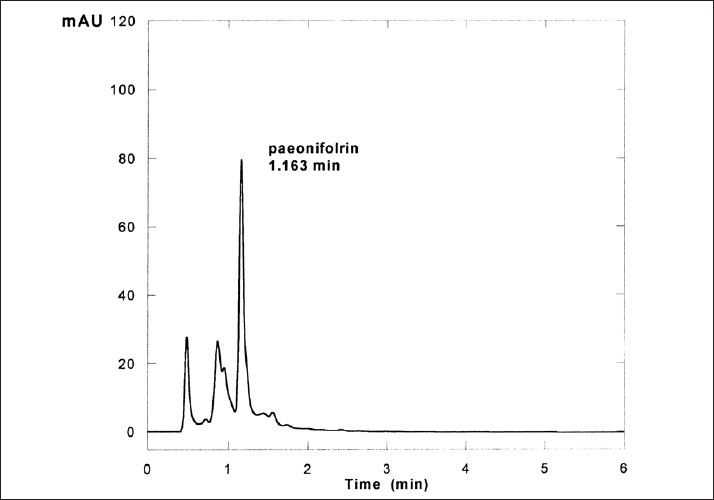
Typical RRLC chromatogram of *Paeonia sinjiang K. Y. Pan*

RRLC conditions: Operation of the RRLC separation and the data processing system were performed by the Agilent Cessation for LC 3D Systems software. Chromatographic separations were achieved using a Zorbas XDB-C_18_ Column with a 1 cm long Guard column. Mobile phase consisted of HPLC grade methanol and 0.05 mol/l of potassium phosphate monobasic buffer at the volume ratio of 38:62 was filtered through a 0.45 μm thick membrane and degassed by ultrasonication under vacuum situation before use. Flow rate was maintained at 1.0 ml/min and samples were monitored with UV detector at the wave length 230 nm. Injection volume was set at 5 μl. RRLC experiments were run at the temperature 30°C. Figure 2 (a typical RRLC Chromatogram of Paeoniflorin) and Figure 3 (a typical RR LC Chromatogram of *Paeonia sinjiang K. Y. Pan*.) were obtained. Using the peak area of the reference in Figures 2 and 3, Paeoniflorin concentration and total substances concentration in *Paeonia sinjiang K. Y. Pan*. were determined. The mean concentration of paioniflorin in *Paeonia sinjiang K. Y. Pan* sample was 4.71. Three parallel sample solutions of Paeoniflorin were prepared. Precision was assessed by injecting each of these three solutions in sequence, one injection for each, which was found complying with experimental requirement at RSD=1.48 %.

### Method development and optimization of stability indicating assay method

The RRLC method was optimized to be applied both *in vitro* studies of paeoniflorin formulation and concentration estimation in biological samples. The main objective of the chromatographic method is to obtain rapid determination of Paeoniflorin concentration in both *Paeonia sinjiangensis K. Y. Pan* and in naturally occurring biological samples. Biological samples were run using C_18_ bonded column or cyano bonded silica column, mobile phases made up of buffers such as phosphate, sulfate and acetate salts with pH controlled between 2 and 7 and organic modifiers such as acetonitrile and methanol were used. RRLC separation result was achieved acceptably only in these chromatographic conditions. It was proved that the isocratic RRLC run using 45% acetonitrile as organic modifier in the mobile phase was successful at separating different components in *Paeonia sinjiangensis K. Y. Pan* and biological samples.

### Method validation

Method validation was achieved by using paeoniflorin reference[14,15] for determination of paeoniflorin content in the *Paeonia sinjiangensis K. Y. Pan*. Validation criteria such as linearity, detection limit, quantification limit, precision, accuracy and specificity were demonstrated in this research.

### Precision

Repeatability (within day): The precision of the analysis was evaluated by carrying out six assays of about 0.5 mg/ml paeoniflorin sample solutions, using linearity regression equation obtained from a series of paeoniflorin standard reference solutions. RSD of the six assays was calculated.

Intermediate precision (inter-day) was assessed by different analysts from the same laboratory. It was performed by quantifying paeoniflorin content of six tablets samples by using standard reference linearity equation. RSD was calculated. Precision tells how well the experiments were controlled and of their reproducibility. It expresses the agreement between a series of measurements obtained from multiple sampling of the same homogeneous sample under prescribed conditions. RSD values for the precision study were 0.4% (inter-day precision) and 0.3% (intra-day precision) for paeoniflorin. This confirms the good precision of the method.

### Linearity of response

Linear calibration plot for this method was obtained over the calibration ranges tested, e.g. ranging from 0.5 to 2.5 mg /ml for paeoniflorin, correlation Coefficient (CC) of this linearity regression equation was greater than 0.999 for the compound. It established an excellent linearity correlation between the peak area and paeoniflorin concentration within this dynamic range. The regression equations: A= 423.4584C-1.3409 (*r* =0.99999, *n* = 6) for paeoniflorin. A = a C + b, where A is the peak area of paeoniflorin, a is the slope, b is the intercept and C is the concentration of paeoniflorin in ug/ml.

### Accuracy (recovery study)

Recovery rate was evaluated by preparing six sample solutions of different concentrations; expected concentration was 0.5 mg/ml. The recovery rate of paeoniflorin was 99.8%.

### Limit of detection and limit of quantification

Recovery rate was also evaluated by introducing limit of detection (LOD) and limit of quantification (LOQ). The LOD and LOQ of paeoniflorin were determined at a signal-to-noise ratio of 3:1, by injecting a series of diluted solutions with known concentration. The LOD of paeoniflorin was 0.2 ug/ml for 5 ul repeated injections. The LOQ of paeoniflorin was 5 ug/ml of 5 ul sample injection volume.

## DISCUSSION

A simple, rapid, effective, binary resolution liquid chromatographic isocratic method was developed for the determination of paeoniflorin. The method was validated and found to be rapid, precise, accurate and linear for the concentration detection and therefore can be used to quantify paeoniflorin content in *Paeonia sinjiang K.Y. Pan*.

Fast experiments running time, confident level in analytical results and cost-effectiveness are the key objectives for today's analytical laboratories. The Agilent 1200 Series Rapid Resolution LC (RRLC) system is designed to meet these challenges by delivering significantly faster results with higher data quality. On one system, RRLC performance can be pushed to new limits and the conventional methods can be continued to run. The plus in separation power and detection capabilities with new innovative system components can be discovered, providing a new level of information about the sample for more informed decisions in shorter time.
